# Asymmetrically enlarged parietal foramina in a rare case of Goldenhar syndrome with a possible etiopathogenesis

**DOI:** 10.18632/oncotarget.23479

**Published:** 2017-12-20

**Authors:** Lika'a Fasih Y. Al-Kzayer, Shamil Naji Sarsam, Nagham Younus Alhur, Tingting Liu, Yozo Nakazawa

**Affiliations:** ^1^ Department of Pediatrics, Shinshu University School of Medicine, Matsumoto, Nagano, Japan; ^2^ Department of Radiology, Ibn Al-Nafees Hospital, Manama, Kingdom of Bahrain; ^3^ Department of Obstetrics and Gynecology, College of Medicine, Hawler Medical University, Erbil, Iraq; ^4^ Department of Pediatric Hematology/Oncology, Xinhua Hospital, Shanghai Jiaotong University School of Medicine, Shanghai, China

**Keywords:** Goldenhar syndrome, oculoauriculovertebral spectrum, enlarged parietal foramina, assisted reproductive technology embryo, depleted uranium (DU)

## Abstract

Goldenhar´s syndrome (GS) also known as oculo-auriculo-vertebral spectrum (OAVS) is a relatively rare condition. GS is of multifactorial etiology that includes environmental and/or genetic factors, in addition to teratogens that disturb the blastogenesis. A 5-year-old girl from Saudi Arabia, was a member of dizygotic twins conceived by assisted reproductive technology (ART), and born with features of GS. She had asymmetrical face, cleft lip and palate, right microphthalmia and microtia. Radiological imaging showed right maxillary and mandibular bone hypoplasia, asymmetrically enlarged parietal foramina, a persistent midline occipital foramen, abnormal bone arising from occipital bone, extending along tentorium cerebelli, and a lipoma at the right tentorium cerebelli. A rudimentary right eye with dermoid cyst and pseudotumor as well as bilateral atresia of external auditory canals were present. Karyotyping was normal. ART and the risk of manipulation of ovum/embryo, was shown to be associated with multiple gestation and an increased risk of major birth defects. Given that our patient was from Eastern-province close to the South of Iraq, where Gulf wars took place and the reported incidence of birth defects, including orofacial malformation, jumped there to about seven-folds, after war, thus, environmental contamination, and the possible teratogenic effect of depleted uranium could not be excluded. In conclusion, our case of GS, disclosed a rare radiological finding in calvarial anatomy, and asserted that, careful clinical evaluation is recommended in cases of GS. ART fertilization risk along with the possible parental environmental exposure were regarded as the potential cooperators of multifactorial etiology in our case.

## INTRODUCTION

Goldenhar´s syndrome (GS) also known as oculo-auriculo-vertebral spectrum (OAVS) is a relatively rare congenital condition characterized by incomplete development of the head and face structures. GS was first described in 1845 by von Arnt, then in 1952 Maurice Goldenhar reported its characteristic features. In 1963, Gorlin named this syndrome as OAVS. A wide range of anomalies were described in OAVS including unilateral or bilateral facial hypoplasia, ear, and eye malformations. In addition, vertebral, cardiac, renal, and cerebral abnormalities were reported to be part of the spectrum. Ear malformation as a minimal clinical sign, must be present, to raise the index of OVAS suspicion. GS occurs in about 1 in 3500 to 1:5600 live births. Although, the exact cause still unknown, a multifactorial etiology that included interaction of environmental and/or genetic factors, in addition to teratogens, were proposed to disturb the blastogenesis. Some of the reported cases had a positive family history (2%), suggestive of an autosomal dominant/ recessive inheritance [1,2].

GS represents anomalies of structures arise from the embryonic first and second branchial arches, the first pharyngeal pouch, the first branchial cleft, and the primordia of the temporal bone. GS could be resulted from failure of the embryonic blood supply during the critical period from third to the fifth week in the development of the craniofacial structures. Although this circulatory failure occurs before the presence of ossification centers in the mandible and maxilla, it could cause abnormal bone formation [3]. Poswillo produced the craniofacial malformation in mice by administering teratogens that caused a hematoma of the stapedial artery and the artery of the second arch and led to necrosis, resulted in a wide spectrum of facial anomalies based on the extent of tissue injury and its ability to regenerate [4].

The purpose of this paper is to report rare cranial anomalies among the growing list of associated and underestimated abnormalities of GS, and to emphasize the multifactorial nature of this syndrome in our case.

## CASE REPORT

A 5-year-old girl from Saudi Arabia who was diagnosed as a case of GS, referred on March 2016, to the radiology department in Ibn Al-Nafees Hospital in Manama- Kingdom of Bahrain, by a maxillofacial surgeon for a computed tomography (CT) scan of the head and neck. Our patient is the only live birth child of a young non-related couple, who tried to conceive again after 3 years following a spontaneous first-trimester miscarriage, and an ectopic pregnancy, using medications. Given the 28-year old mother had polycystic ovary and a troublesome medical history, an intracytoplasmic sperm injection (ICSI) procedure was done and (3 embryos were transferred). Our case was the living member of a dizygotic twins whilst the other member of the twins was miscarried at the 11th week of gestation. The mother had a long history of hypochromic microcytic anemia with a hemoglobin range of (7-9 gm/dl), and supposed to be a carrier of alpha thalassemia. During the 12th gestational week of our case, the mother had progressive pallor and her hemoglobin dropped down to (6 gm/dl), necessitated blood transfusion. Then at the 26th week, the mother had signs of preterm labor, which was halted using medication, and the pregnancy was ended by Cesarean section. Of note, the mother who was allergic to a long list of drugs, received hormonal therapy to trigger or regulate ovulation, drugs before and after performing ICSI, then during pregnancy, she received folic acid 5mg/day, and calcium was started from the 4th month of pregnancy. Soon after delivery, the mother who had 5 years history of migraine and tingling sensation in her limbs, developed an epileptic attack and proved to have multiple sclerosis. There was no gestational diabetes, no history of smoking, and no family history of craniofacial malformations. According to the declaration of Helsinki, written informed consent was obtained from the parents of the patient for publication of this Case report and the accompanying images.

**Figure 1:**
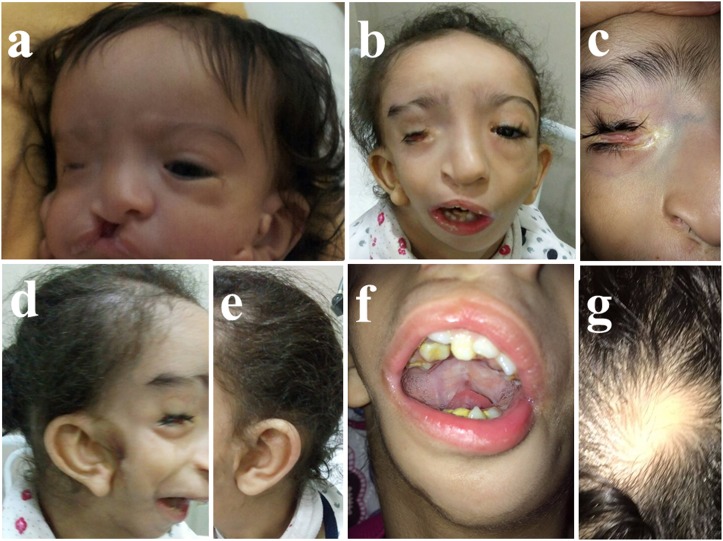
Craniofacial anomalies **a.** At 2 months, right microphthalmia, cleft lip/palate on the right side and bilateral preauricular rudimentary ear tags (before repair). **b.** At 5 years-old, asymmetrical face with underdevelopment of the right side of the face muscles, macrostomia and micrognathia. **c.** Right microphthalmia with palpebral fissure. **d.** and **e.** Right grade I microtia (misshapen ear), bilateral preauricular rudimentary ear tags (after operation). **f.** Crowded teeth with caries, and abnormal tongue shape. **g.** The skin over the calvarial parietal bone defect with no hair and localized soft tissue beneath it.

**Figure 2:**
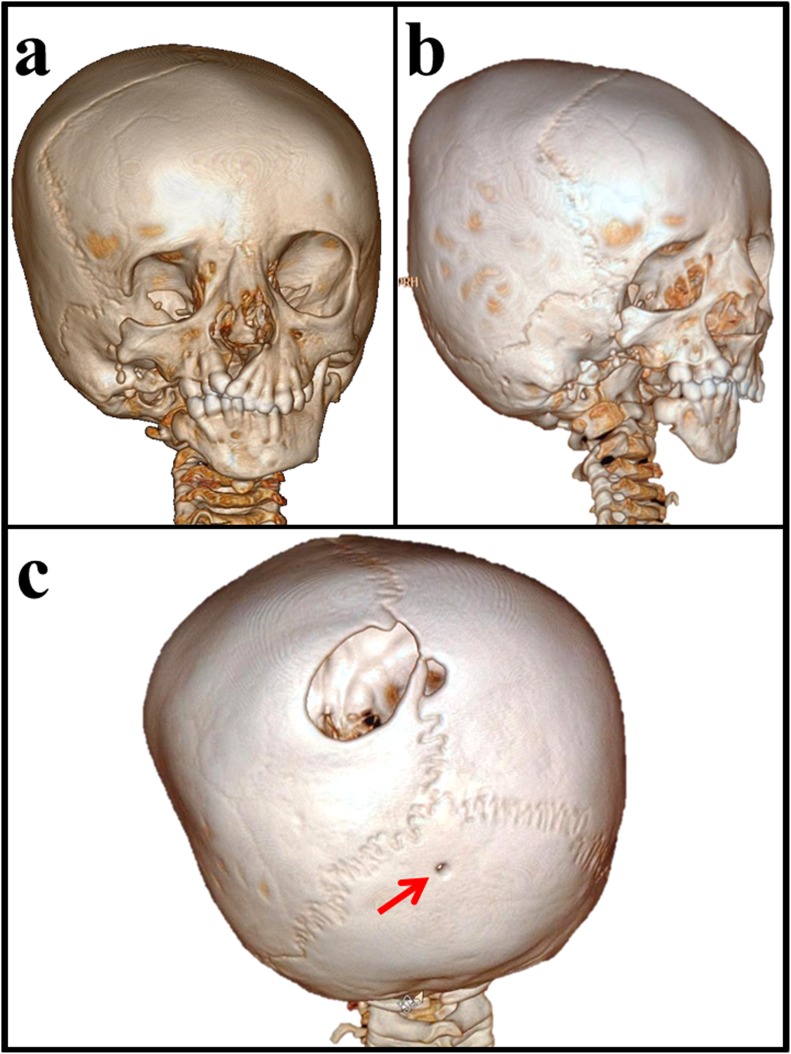
Three dimensional-CT scan of the skull **a.** Asymmetrical facial bones, small right orbit with a small sphenoid bone. Hypoplastic right zygomatic bone with missing of the frontal process, temporal process and the zygomatic arch. **b.** Hypoplastic right maxilla and temporal bone with missing of the right styloid process and cleft palate, in addition to missing of mandibular condyle, coronoid process, ramus and part of the body of the right mandible. **c.** Bilateral parietal osseous defects, represented asymmetrically enlarged parietal foramina, larger on the right side (3 × 2.5 cm) reaching the sagittal suture. A persistent occipital foramen at midline of occipital bone (*arrowed*).

**Figure 3:**
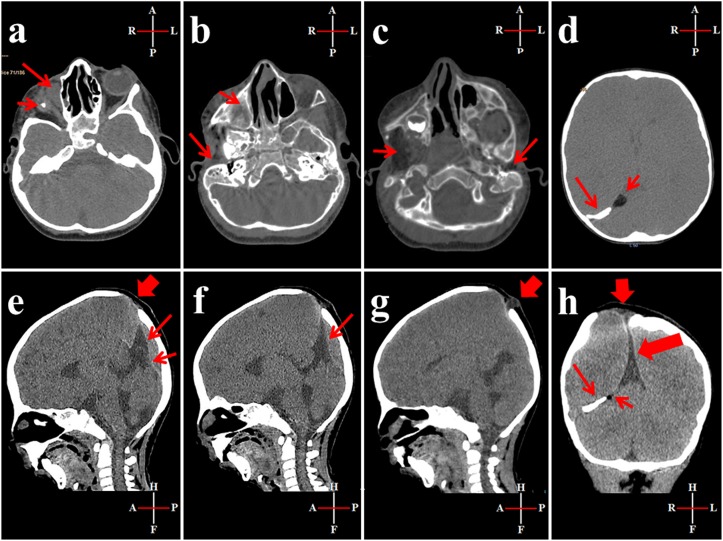
CT scan of the head **a.** *Short arrow:* soft tissue lesion (1 cm) with a small central calcified focus at the right orbit suggestive of a rudimentary eye with a dislocated lens or a dermoid cyst. *Long arrow:* oval soft tissue lesion (1 × 1.5 cm) at the medial wall of the right orbit, in the site of origin of inferior oblique muscle, could be a pseudotumor. **b.** *Short arrow:* non-aerated right maxillary sinus. *Long arrow:* atresia of the right external auditory canal. **c.** *Short arrow:* atrophied right pterygoid muscles. *Long arrow:* atresia of the left external auditory canal. **d.** *Short arrow:* a small lipoma at the right tentorium cerebelli. *Long arrow:* abnormal bone arising from the inner table of the right occipital bone and extending along the tentorium cerebelli. **e.** *Sagittal view; wide arrow:* large parietal foramen. *Long arrow:* tenting of superior cerebellar cistern, *short arrow:* straight sinus. **f.** *Sagittal view; arrow:* tenting of superior cerebellar cistern reaching near the right parietal bony defect. **g.** *Sagittal view; arrow:* small meningocele arising through the right parietal foramen. **h.** *Coronal view; wide short arrow:* small meningocele arising through the right parietal foramen, *wide long arrow:* tenting of superior cerebellar cistern. *Long thin arrow:* abnormal bone arising from the inner table of the right occipital bone and extending along the tentorium cerebelli. *Short thin arrow:* small lipoma at the right tentorium cerebelli.

After birth, the full term baby who was diagnosed antenatally to have cleft lip and palate, was found to have dysmorphic features and intrauterine growth restriction. Due to upper airway obstruction related to her craniofacial malformation, mechanical ventilation and tracheostomy tube were used for a while. After being stabilized, she was referred to ENTist for repairing cleft lip at 6 months of age, and cleft palate and preauricular ear tags at 18 months. Then she was referred to ophthalmologist, pediatric and plastic surgeons, and maxillofacial specialist for intervention. As shown in Figure 1, the patient had features of craniofacial anomalies including microcephaly, asymmetrical face, right microphthalmia, but normal left eye with preserved vision. She had mandibular and maxillary hypoplasia, in addition to cleft lip and palate in the right side, macrostomia to right side, micrognathia, malocclusion, crowded teeth with caries, difficulties of mastication, and abnormal tongue shape. Right grade I microtia, bilateral preauricular rudimentary ear tags and bilateral membranous ear canals. As shown in Figure 2a and 2b, the three dimensional-CT scan of the skull revealed asymmetrical facial bones, and a small right orbit. Bilateral parietal osseous defects, represented asymmetrically enlarged parietal foramina were found, being larger on the right side, in addition, a persistent midline occipital foramen, was seen (Figure 2c). Figure 3a-3c, showed a right rudimentary eye with a dislocated lens or dermoid cyst, and an oval soft tissue lesion interpreted as a pseudotumor. Abnormal bone arising from the inner table of the right occipital bone and extending along the tentorium cerebelli was detected, and a small lipoma at the right tentorium cerebelli was seen (Figure 3d and 3h). Bilateral atresia of external auditory canals were detectable, and right abnormal middle ear with small size ossicles mainly the malleus, whereas inner ear was normal on both sides. Through the big right parietal foramen, a slight herniation of the underlying meninges was evident, to conform to the defect, connected to the superior cerebellar cistern (Figure 3e-3h). Systemic examination revealed that the child is mentally subnormal with delayed milestones, slurred speech and retarded growth, in addition to the presence of conductive hearing loss. The examination of chest, heart, and abdomen, was unremarkable with the presence of normal female genitalia. Gastroesophageal reflux was diagnosed on a clinical basis. Spines, hips, and limbs were normal with no deformity in accordance to clinical along with radiological evaluation. Ultrasound of the abdomen and echo of the heart were both normal and karyotyping was normal as well.

## DISCUSSION

Our case has the characteristic features of GS with the additional presence of calvarial bone defects. Although skeletal defects were reported in most cases of GS, calvarial bone defects and parietal bone in particular, was rarely described. Given the accessible published literatures since the 1960s, we could find 2 cases of parietal bone defects, whereas enlarged parietal foramina and a persistent occipital foramen were not reported (Table 1) [5–7]. Parietal foramina are a normal feature of fetal skull development, located close to the intersection of the sagittal and lambdoid sutures, caused by deficient ossification around the parietal notch, which is normally obliterated around the 20th week of fetal life. Through parietal foramina the parietal emissary vein passes and drains into the superior sagittal sinus. Small foramina are sometimes even considered to be part of the spectrum of normal variation. Its bilateral incidence is 20%-62% (Boyd, 1930; Berry and Berry, 1967; Hauser and De Stefano, 1989) [8]. The abnormal bone found in our case arising from the occipital bone with the lipoma could somewhat resemble that reported by Wilson (in patient-2) [5].

**Table 1:** A comparison of our case and two previously reported cases of parietal bone defects in Goldenhar syndrome or Oculo-auriculo-vertebral spectrum

	Reference	Al-Kzayer et al. [Current report]	Mellor et al. [6]	Cohen [7]
	Year - Case number	2017 - Our case	1973 - Case 2	1971 - Case 3
Sex		Female	Female	Male
Family history		Unremarkable	Unremarkable	?
Consanguineous marriage		Non	Non	?
Mother health				
	Medical history	Hypochromic microcytic anemia (alpha thalassemia carrier?), received blood at 12th week of pregnancy, multiple sclerosis	Healthy	?
	Gynecologic/Obstetric	Polycystic ovary syndrome, miscarriage, ectopic pregnancy, conceived with our case by ICSI^a^	Normal/ uncomplicated pregnancy	?
Birth status		Born by Caesarean section at wk 37, birth weight: 1860 g, length: 44cm, head circumference: 30cm, 3 days on mechanical ventilation	Normal vaginal delivery at week 41, birth weight: 3600 g, Apgar score was 3 at 1 min, tracheostomy at 10 min	?
Karyotype		(46, XX)	(46, XX)	?
Craniofacial abnormalities				
	Main side of defects	R^b^	R	?
	Face	Asymmetrical, hypoplasia of the muscles of face on R, flat nasal bridge	Asymmetrical, hypoplasia of face on R side	Asymmetrical
	Skull	Microcephaly, hypoplasia of R maxillary, R mandibular and R zygomatic bones, hypoplastic R temporal and R sphenoid bone and small R orbit, asymmetrically enlarged parietal foramina (bigger in R side), persistent occipital foramen, abnormal bone arising from inner table of R occipital bone extending along tentorium cerebelli	R maxillary hypoplasia, R mandibular hypoplasia, skull x-rays revealed a large parietal bony defect and a smaller one in the frontal region	Cranial asymmetry, parietal osseous defect
	Ear	Grade I R microtia, bilateral periarticular rudimentary ear tag and bilateral atresia of external auditory canals, R small size ossicles (malleus) and conductive hearing loss	Hypoplastic/ malformed R ear, atresia of R external auditory meatus, and L^c^ preauricular appendix	Malformed ears
	Eye	R microphthalmia, rudimentary R eye, a dermoid cyst in R eye and pseudotumor	R lipodermoid cyst, coloboma of L upper eyelid	Unilateral anophthalmia
	Oral	R cleft lip/palate, macrostomia, malocclusion, crowded teeth with caries, abnormal tongue shape	Absent R half of tongue, cleft palate, epiglottis and laryngeal inlet were very small and deviated to L.	Cleft lip/palate, micrognathia
Central nervous system		Meningocele bulging through the R parietal foramen connected to superior cerebellar cistern, small lipoma at R tentorium cerebelli, mentally subnormal	?	Mental retardation
Vertebral		Non	Multiple hemivertebrae of the cervical spines	Non
Cardiac		Non	Patent ductus arteriosus	Non
Gastrointestinal tract		Gastroesophageal reflux	?	?
Renal		Non	?	?
Others		Hypereactive airways, snoring, recurrent chest infection	Torticollis, recurrent chest infection, died at 5 weeks of age	?

^a^Intracytoplasmic sperm injection.

^b^Right.

^c^Left.

### Etiopathogenesis in our case

#### Assisted reproductive technology

Assisted reproductive technology (ART), such as ICSI and the risk of manipulation of ovum and embryo, was shown in general to be associated with multiple gestation and an increased risk reaching up to 10% for major birth defects in some studies compared to naturally conceived embryos [9]. Our case, was a product of dizygotic twin from ICSI, thus, there is a possible predisposing factor related to the ART. Yovich J L, et al, reported one case of GS among 36 cases of ART embryos in a triplet pregnancy [10]. Johnson J M, et al, and Kokavec R, et al, reported cases of GS resulted from ART pregnancies, with the latter being in a dizygotic twin as our case [11, 12]. Taken together, Mastroiacovo et al. (1999), described an increase of at least 60% in the risk of total malformations for twins compared with singletons in three registries. In OAVS there are some arguments that twinning may increase the risk for OAVS, or that twinning and OAVS have a common basis [13].

#### Environmental factors

Our patient is of Saudi nationality, living in the Eastern province, Al-Qateef city, which borders the Arabian- Persian Gulf, and it is the place for most of Saudi Arabia's oil production and global hub for chemical industries. The Eastern province had reported the second highest frequency of birth defects, including GS next to the capital Al-Riyadh, together they accounted for about half of cases of birth defects in the country [14]. Taken together, the Eastern province is close to Basra; a city in the South of Iraq, which was heavily bombarded with depleted uranium (DU) since the first Gulf War (GW) 1991 [15]. Knowing that DU has a radioactive half-life measured in billions of years and toxic effects through air, water, and soil contamination, thus, spreading environmental pollution is an inevitable consequence. Hence, a possible relationship between DU teratogenicity and GS has been suggested based on previously published reports [16]. In Basra, the reported incidence of birth defects, including orofacial malformation among 1000 births jumped from 3.04 to 22.19 in the years 1990 and 2001, respectively. Similarly, in other Iraqi report, the skeletal abnormalities rose from 2.8% to 4.6% for the periods (1989-1990) and (1992-1993), respectively [16]. Likewise, Araneta MR, et al [17], 1997, reported a three-fold increase in the risk of GS among children born to GW veterans, but the association was not statistically significant. Given the rarity of GS, the sample size of 75,414 births was insufficient to determine whether the observed excess rate in children of GW veterans was just a matter of chance. Thus, the environmental contamination has a potential impact related to parental exposure and the birth of offspring with GS in our case.

## CONCLUSION

To the best of our knowledge this is the first report of GS presenting with bilateral irregularly and asymmetrically enlarged parietal foramina and a persistent occipital foramen in addition to the presence of the abnormal bone arising from the occipital bone. Accordingly, careful imaging and clinical evaluation are recommended in cases of GS. ART fertilization risk along with the possible parental environmental DU exposure risk, were regarded as the top potential cooperators of multifactorial etiology in our case.

## Abbreviations

GS: Goldenhar´s syndrome
OAVS: Oculo-auriculo-vertebral spectrum
ART: Assisted reproductive technology
CT: Computed tomography
ICSI: Intracytoplasmic sperm injection
DUI: Depleted uranium
GW: Gulf War
